# PfAgo-Enhanced LAMP Assay for Rapid and Specific Detection of *Vibrio alginolyticus* and *Vibrio vulnificus*

**DOI:** 10.3390/foods15142506

**Published:** 2026-07-15

**Authors:** Changzheng Shi, Lifang Hao, Zhaoxin Lu, Xiaomei Bie

**Affiliations:** College of Food Science and Technology, Nanjing Agricultural University, Nanjing 210095, China

**Keywords:** LAMP, Pyrococcus furiosus Argonaute (PfAgo), pathogen detection, seafood safety, two-step workflow

## Abstract

Rapid and accurate detection of *Vibrio alginolyticus* and *Vibrio vulnificus* is important for seafood safety and foodborne infection control. In this study, we developed a detection platform that combines loop-mediated isothermal amplification (LAMP) with Pyrococcus furiosus Argonaute (PfAgo)-mediated sequence-specific cleavage. The LAMP rapidly amplified pathogen-specific target sequences under isothermal conditions, while PfAgo provides an additional layer of sequence verification through a programmable guide DNA-directed cleavage step to reduce false-positive signals caused by nonspecific amplification. Under optimized conditions, the assay achieved limits of detection of 3 × 10^1^ CFU·mL^−1^ for *V. alginolyticus* and 10^2^ CFU·mL^−1^ for *V. vulnificus*, with no positive signals observed among the tested non-target bacteria. In artificially contaminated salmon samples, both *V. alginolyticus* and *V. vulnificus* were detected without enrichment after simple boiling-based DNA extraction, with a practical detection limit of 2.9 × 10^2^ CFU·mL^−1^ in the final homogenate. Compared with culture-based methods and instrument-intensive molecular assays, the proposed LAMP-PfAgo platform exhibits a rapid two-step workflow with an added sequence-verification layer. These results support LAMP coupled with Argonaute-mediated cleavage as a practical strategy for foodborne pathogen monitoring in seafood.

## 1. Introduction

*Vibrio alginolyticus* (*V. alginolyticus*) and *Vibrio vulnificus* (*V. vulnificus*) are important halophilic bacteria that are widely distributed in marine and estuarine environments. Both pathogens threaten aquaculture by causing mortality in aquatic organisms and substantial economic losses [1]. *V. alginolyticus* can cause wound infections in humans, which may progress to severe disease, and has therefore attracted increasing attention [2]. *V. vulnificus* is recognized as one of the most virulent foodborne pathogens and can cause septicemia and necrotizing wound infections with high mortality rates [3]. Both species also carry virulence-associated genes and show increasing antimicrobial resistance, further complicating seafood safety management [4,5]. Rapid and sensitive detection methods are therefore needed for seafood safety control.

The high genetic relatedness and similar phenotypes of *Vibrio* species, particularly between *V. alginolyticus* and *V. vulnificus*, can lead to misidentification and delays in timely clinical or food-safety interventions [6]. Culture-based methods are reliable but time-consuming, whereas immunological assays require specific antibodies and may be less suitable for early and rapid pathogen detection [7]. Loop-mediated isothermal amplification (LAMP) can detect target genes under constant-temperature conditions, but conventional LAMP results often rely on gel electrophoresis or visual observation of precipitation. These results can be subjective, contamination-prone, or vulnerable to false-positive signals, indicating the need for a more specific detection workflow [8].

Clustered regularly interspaced short palindromic repeats (CRISPR) and CRISPR-associated proteins (Cas) provide highly specific nucleic acid recognition and have been combined with LAMP to improve assay specificity. For example, LAMP-CRISPR-Cas12a has been used for specific detection of Escherichia coli O157:H7 with ultraviolet (UV)-based visual readout, reducing reliance on complex instruments and mitigating false-positive signals [9]. However, CRISPR-Cas assays still face practical constraints. Cas13a-based detection requires an additional transcription step to convert DNA into RNA, increasing assay complexity, cost, and time [10]. Cas12a can directly target double-stranded DNA, but its activity depends on protospacer adjacent motif (PAM) sequences, restricting target-site selection and assay flexibility [11]. In addition, Cas proteins can be sensitive to reaction conditions, which may affect robustness in resource-limited on-site testing [12]. Alternative nucleic acid recognition tools that are compatible with LAMP therefore remain valuable.

Argonaute (Ago) proteins are programmable nucleases that specifically recognize and cleave nucleic acid targets [13]. Compared with CRISPR-Cas systems, Ago proteins do not require PAM sequences, thus offering greater flexibility in target site selection. They use short guide DNAs (gDNAs) that are stable, easy to design, and readily synthesized, which reduce the off-target risks [14]. Pyrococcus furiosus Argonaute (PfAgo) from the hyperthermophilic archaeon Pyrococcus furiosus has been widely explored for nucleic acid detection because of its thermostability and high cleavage efficiency. In PfAgo-based assays, amplified nucleic acid fragments can be recognized by PfAgo-gDNA complexes, leading to probe cleavage and fluorescence signal release [15]. Recent studies have demonstrated that the integration of specific primers, exonuclease I, and PfAgo enables one-step cleavage, facilitating ultrasensitive detection of foodborne pathogens. This assay enabled rapid and sensitive detection of *Salmonella Typhi* and *Staphylococcus aureus* in actual food samples, highlighting the potential of PfAgo-based strategies for rapid foodborne pathogen screening [16]. Because LAMP and PfAgo-mediated cleavage can be integrated in a sequential workflow, their combination provides a direct route for target amplification followed by sequence-specific verification [17].

Here, we established a rapid and specific LAMP-PfAgo assay for *V. alginolyticus* and *V. vulnificus*. We optimized key parameters of the LAMP and PfAgo cleavage modules, evaluated analytical specificity and sensitivity, and tested practical applicability using artificially contaminated salmon samples. This work addresses the limitations of conventional methods and CRISPR-Cas based detection assays by developing a two-step LAMP-PfAgo detection workflow for *V. alginolyticus* and *V. vulnificus*.

## 2. Materials and Methods

### 2.1. Bacterial Strains and Genomic DNA Extraction

The standard strain and isolates of *V. alginolyticus* and *V. vulnificus* were used as target strains in this study. The biotypes of the tested *V. vulnificus* strains were not further determined in the present study. Several non-target bacterial species, including other *Vibrio* species and common foodborne pathogens, were used to evaluate the specificity of the developed assay. All strains used in this study are summarized in Table 1. All strains were maintained in 40% glycerol stocks at −80 °C in our laboratory. Before use, bacterial strains were streaked onto Luria–Bertani (LB) agar plates and incubated at 37 °C for activation. A single colony was subsequently inoculated into LB broth and cultured overnight at 37 °C with shaking. All *Vibrio* strains were cultured in LB medium supplemented with 3% (*w*/*v*) NaCl.

Genomic DNA was extracted as described previously [18]. Briefly, 1 mL of bacterial culture was centrifuged at 12,000× *g* for 5 min. The pellet was resuspended in 100 μL TE buffer, boiled in a water bath at 100 °C for 10 min, and immediately placed on ice for 10 min. After centrifugation at 12,000× *g* for 5 min, the supernatant containing genomic DNA was collected and stored at −20 °C until use.

### 2.2. Design of LAMP Primers, gDNAs, and Probes

Species-specific target genes, N646_RS01740 for *V. alginolyticus* and AOT11_08030 for *V. vulnificus*, were selected based on previous genomic screening in our laboratory. Four LAMP primer sets were designed for each target pathogen using PrimerExplorer V5 software (http://primerexplorer.jp/e/index.html, accessed on 7 November 2024) according to previously established LAMP primer design principles [19,20]. Primer sequences are listed in Table 2 and Table 3.

To establish the PfAgo-mediated cleavage system, three groups of 16 nt gDNAs were designed for each pathogen using SnapGene 6.0.2 software. All gDNAs were synthesized with a 5′-phosphate modification to enable PfAgo loading. Corresponding molecular beacon (MB) probes labeled with fluorescent and quenching groups were also designed for fluorescence signal generation. The sequences of all gDNAs and probes are shown in Table 4 and Table 5. The design strategy was based on the programmable cleavage characteristics of Argonaute-mediated nucleic acid detection systems.

All primers, gDNAs, and molecular beacon probes were synthesized by GenScript Biotech Corporation (Nanjing, China). PfAgo (Cat. No. JY0316-L) was purchased from Tiosbio (Beijing, China). Bst 3.0 DNA polymerase, isothermal amplification buffer, and dNTPs were purchased from New England Biolabs (Ipswich, MA, USA).

### 2.3. LAMP Reaction and Optimization Conditions

The LAMP reaction was performed in a total volume of 25 μL containing outer primers (F3/B3, 0.2 μmol/L each), inner primers (FIP/BIP, 0.8 μmol/L each), loop primers (LF/LB, 0.4 μmol/L each), 1.4 mmol/L dNTPs, 6.0 mmol/L MgSO_4_, 1× isothermal amplification buffer, 0.32 U/μL Bst 3.0 DNA polymerase, 2 μL genomic DNA template, and nuclease-free water to volume. Amplification was initially conducted at 65 °C for 40 min, consistent with commonly reported LAMP conditions for foodborne pathogen detection [8].

To improve amplification efficiency, key reaction parameters were systematically optimized, including Mg^2+^ concentration (2.0–10.0 mM), dNTP concentration (1.0–1.8 mM), amplification temperature (60–65 °C), Bst 3.0 DNA polymerase dosage, and inner-to-outer primer ratio (2:1–10:1). During optimization, only one parameter was varied at a time while all other reaction conditions were kept constant. Amplification performance was evaluated according to fluorescence intensity and threshold cycle (Ct) values obtained from real-time fluorescence monitoring.

### 2.4. Optimization of PfAgo-Mediated Cleavage Reaction Conditions

The PfAgo cleavage reaction was performed in a total volume of 20 μL containing 2 μL PfAgo protein (Tiosbio, JY0316-L), 1 μL gDNA, 0.5 μL Mn^2+^ solution, 2 μL 10× PfAgo Endonuclease Reaction Buffer (supplied with the commercial PfAgo protein), 1 μL molecular beacon probe, 2 μL LAMP product, and nuclease-free water to final volume. The final concentrations of gDNA, Mn^2+^, and molecular beacon probe were initially set at 0.5 μM, 1 mM, and 0.5 μM, respectively.

Following LAMP, the PfAgo-assisted nucleic acid detection step was performed as previously described with minor modifications [17]. In total, 2 μL of the LAMP product was transferred to the PfAgo cleavage reaction mixture. The PfAgo-mediated cleavage reaction was then incubated at 95 °C for 30 min, followed by fluorescence measurement. The fluorescence signal was generated through a target-dependent PfAgo cleavage cascade. Briefly, 5′-phosphorylated gDNAs guided PfAgo to cleave the LAMP amplicons at specific sites. The resulting short DNA fragments served as secondary guides to direct PfAgo-mediated cleavage of the molecular beacon probe, separating the fluorophore from the quencher and producing fluorescence signals [21].

The PfAgo cleavage system was further optimized by varying gDNA concentration (0–1.0 μmol/L), PfAgo protein dosage, Mn^2+^ concentration (0–1.0 mmol/L), and cleavage reaction time (5–30 min). All optimization experiments were performed under otherwise identical conditions, and fluorescence intensity was used as the primary evaluation indicator.

### 2.5. Specificity and Sensitivity Evaluation

The specificity of the developed LAMP-PfAgo assay was evaluated using genomic DNA extracted from target strains and non-target bacterial species listed in Table 1. Nuclease-free water was used as the negative control. Cross-reactivity within the tested bacterial panel was assessed according to fluorescence signal generation after amplification and PfAgo cleavage.

To determine the limit of detection (LOD), overnight cultures of *V. alginolyticus* and *V. vulnificus* were serially diluted 10-fold in sterile saline. Bacterial concentrations were determined by plate counting, and genomic DNA extracted from each dilution was used as the template for the optimized LAMP-PfAgo assay. The lowest bacterial concentration that consistently produced detectable fluorescence signals in three independent experiments was defined as the limit of detection (LOD), according to previously reported molecular diagnostic evaluation criteria [22].

### 2.6. Preparation of Artificially Contaminated Samples

Artificial contamination experiments were performed to evaluate the practical applicability of the LAMP-PfAgo method for detecting *V. alginolyticus* and *V. vulnificus* in food samples. Briefly, 22.5 g of salmon was placed into a stomacher bag containing 225 mL of alkaline peptone water (APW). Target bacterial suspensions were prepared at approximately 100-fold higher concentrations than the intended final contamination levels, with the highest prepared suspension concentration of approximately 10^8^ CFU·mL^−1^. For each contamination level, 2.5 mL of bacterial suspension was added to the corresponding sample to obtain final bacterial concentrations ranging from approximately 10^6^ to 10^0^ CFU·mL^−1^ in the homogenates. After homogenization, 1 mL of each homogenate was collected for genomic DNA extraction and tested using the established LAMP-PfAgo method.

### 2.7. Statistical Analysis

All experiments were performed in triplicate, and the results are presented as mean ± standard deviation (SD). Statistical analysis and graphical visualization were performed using GraphPad Prism 8.0 software (GraphPad Software, Boston, MA, USA). Differences among groups were analyzed by one-way analysis of variance (ANOVA), with *p* < 0.05 considered statistically significant. Different lowercase letters indicate significant differences among groups.

## 3. Results

### 3.1. Optimization of the LAMP System

**Figure 1:**
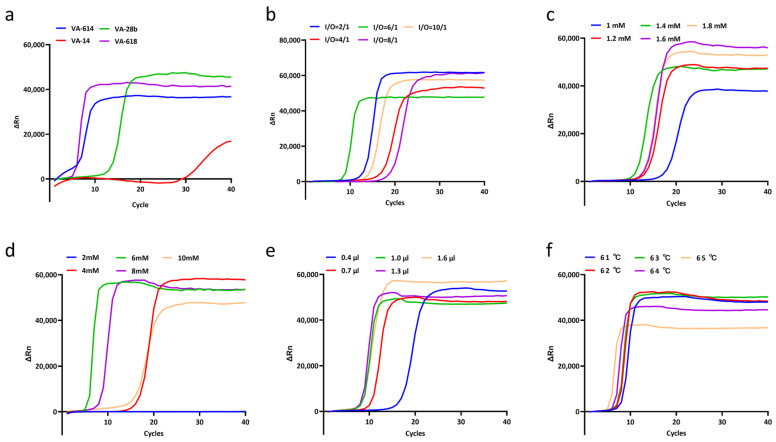
Optimization of the LAMP system for *V. alginolyticus*. Real-time fluorescence amplification curves were used to optimize the LAMP reaction conditions for *V. alginolyticus*. (**a**) Screening of four LAMP primer sets. (**b**) Optimization of the inner-to-outer primer ratio. (**c**) Optimization of dNTP concentration. (**d**) Optimization of Mg^2+^ concentration. (**e**) Optimization of Bst 3.0 DNA polymerase dosage. (**f**) Optimization of reaction temperature. Amplification performance was evaluated according to amplification onset and ΔRn values, and the optimized conditions were selected for subsequent PfAgo-mediated detection.

The LAMP conditions were independently optimized for *V. alginolyticus* and *V. vulnificus* to establish efficient upstream amplification modules for the subsequent PfAgo-mediated detection assay. For *V. alginolyticus*, four primer sets were evaluated using genomic DNA as the template. Among them, VA-618 produced an early amplification curve with a stable fluorescence signal and was selected for further optimization (Figure 1a). The inner-to-outer (I/O) primer ratio was then screened, and an I/O ratio of 6:1 yielded rapid amplification onset with a high ΔRn value (Figure 1b). Further optimization showed that 1.4 mM dNTPs, 6 mM Mg^2+^, and 1.0 μL Bst 3.0 DNA polymerase supported rapid and stable amplification (Figure 1c–e). Although several temperatures generated detectable amplification, 65 °C provided rapid signal development and was selected as the reaction temperature for the *V. alginolyticus* LAMP assay (Figure 1f).

**Figure 2:**
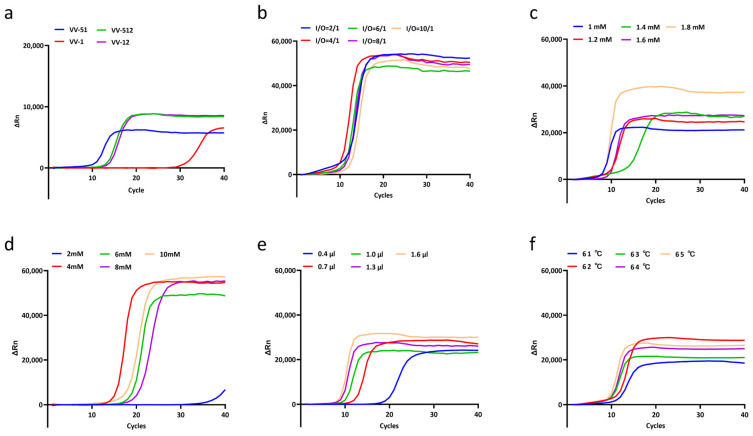
Optimization of the LAMP system for *V. vulnificus*. Real-time fluorescence amplification curves were used to optimize the LAMP reaction conditions for *V. vulnificus*. (**a**) Screening of four LAMP primer sets. (**b**) Optimization of the inner-to-outer primer ratio. (**c**) Optimization of dNTP concentration. (**d**) Optimization of Mg^2+^ concentration. (**e**) Optimization of Bst 3.0 DNA polymerase dosage. (**f**) Optimization of reaction temperature. The optimal reaction parameters were determined based on rapid amplification kinetics and stable fluorescence signals, providing the upstream amplification system for subsequent PfAgo cleavage analysis.

The same optimization strategy was applied to the *V. vulnificus* LAMP system. Primer set VV-51 showed the earliest amplification among the four candidate primer sets and was selected for subsequent assays (Figure 2a). Screening of primer ratios indicated that an I/O ratio of 4:1 produced rapid amplification and a strong fluorescence response (Figure 2b). For the reaction components, 1.8 mM dNTPs and 4 mM Mg^2+^ produced favorable amplification profiles (Figure 2c,d). In the Bst 3.0 DNA polymerase screening, the plotted curves indicate that 1.6 μL supported efficient amplification (Figure 2e). For temperature optimization, 65 °C enabled rapid amplification and was selected for the *V. vulnificus* LAMP reaction (Figure 2f). Together, these results established species-specific optimized LAMP systems for *V. alginolyticus* and *V. vulnificus*, providing reliable amplified products for downstream PfAgo cleavage.

### 3.2. Establishment and Optimization of the LAMP-PfAgo System

**Figure 3:**
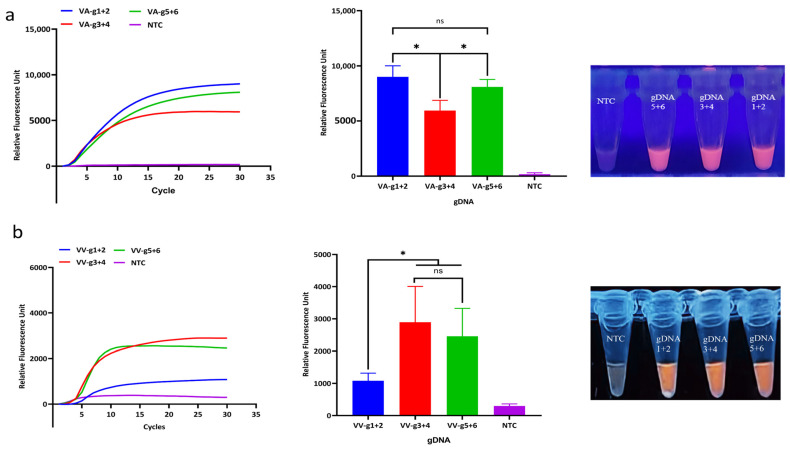
Screening of optimal gDNA combinations for the LAMP-PfAgo cleavage system targeting *V. alginolyticus* and *V. vulnificus*. (**a**) Evaluation of different gDNA combinations for *V. alginolyticus* based on relative fluorescence intensity, and fluorescence visualization under UV illumination. (**b**) Evaluation of different gDNA combinations for *V. vulnificus* based on relative fluorescence intensity. “*” indicates statistically significant differences (*p* < 0.05), and “ns” indicates no significant difference.

To identify the suitable gDNA combinations for PfAgo-mediated cleavage, three candidate gDNA groups were evaluated for both *V. alginolyticus* and *V. vulnificus.* For *V. alginolyticus*, the tested gDNA groups generated distinguishable fluorescence signals relative to the no-template control (NTC) (*p* < 0.05) (Figure 3a). For *V. vulnificus*, the candidate gDNA groups also differed in fluorescence intensity, allowing selection of the group with the strongest signal and clearest separation from the negative control (Figure 3b). These results demonstrated that different gDNA combinations exhibited distinct cleavage efficiencies toward amplified target sequences. Therefore, the VA-gDNA1+2 combination for *V. alginolyticus* and the VV-gDNA3+4 combination for *V. vulnificus* were selected for subsequent optimization experiments.

**Figure 4:**
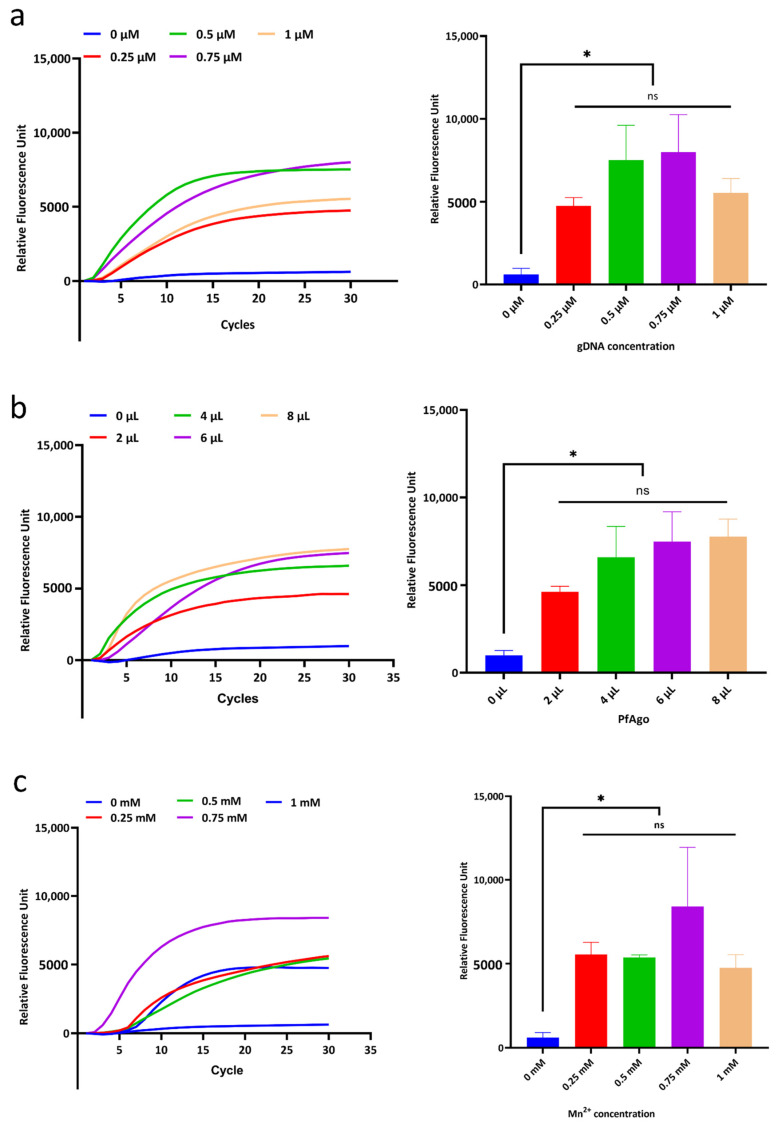
Optimization of the LAMP-PfAgo cleavage system for *V. alginolyticus*. (**a**) Optimization of gDNA concentration. (**b**) Optimization of PfAgo protein dosage. (**c**) Optimization of Mn^2+^ concentration. Amplification curves and corresponding relative fluorescence intensities are shown for each condition. “*” indicates statistically significant differences (*p* < 0.05), and “ns” indicates no significant difference.

The reaction parameters of the PfAgo cleavage system were further optimized to improve fluorescence signal intensity and detection efficiency for both target pathogens. For the *V. alginolyticus* detection system, fluorescence intensity increased with increasing gDNA and Mn^2+^ concentrations and reached maximum values at 0.75 μM gDNA and 0.75 mM Mn^2+^, respectively (Figure 4a,c). The highest fluorescence signal was obtained with 8 μL PfAgo protein (Figure 4b). Thus, the optimized PfAgo cleavage conditions for *V. alginolyticus* were 0.75 μM gDNA, 0.75 mM Mn^2+^, and 8 μL PfAgo protein.

**Figure 5:**
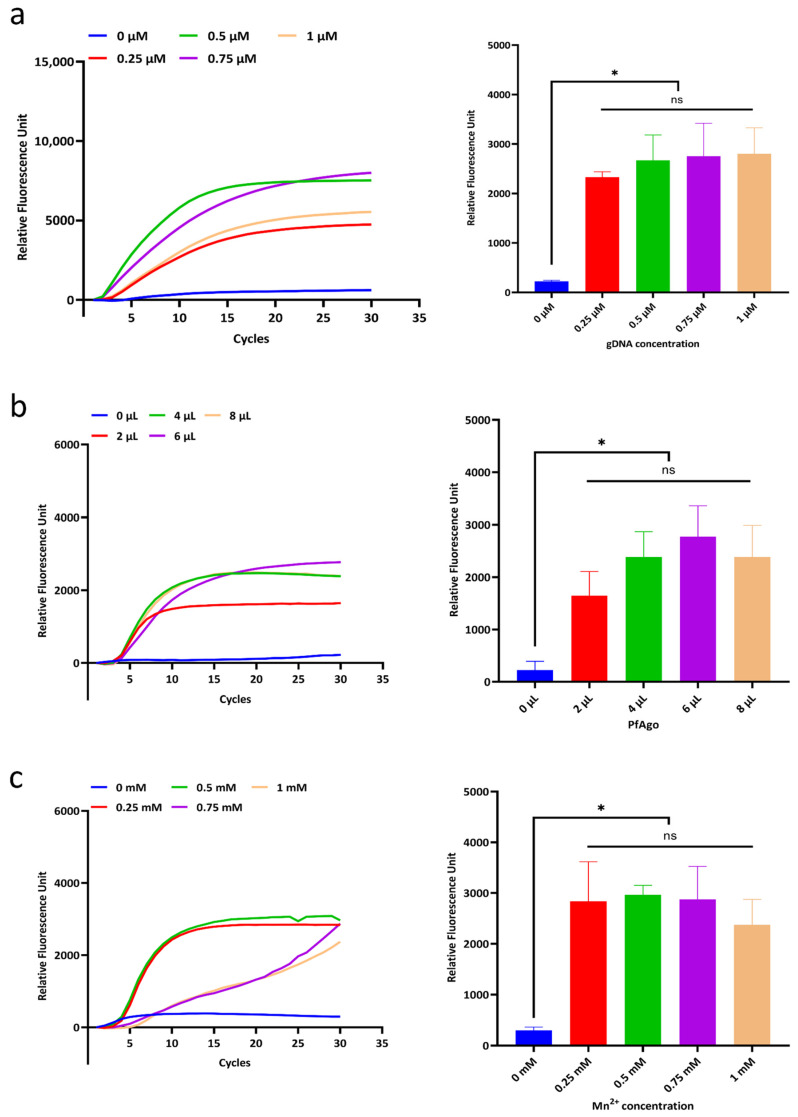
Optimization of the PfAgo-mediated cleavage system for *V. vulnificus*. (**a**) Optimization of gDNA concentration. (**b**) Optimization of PfAgo protein dosage. (**c**) Optimization of Mn^2+^ concentration. Amplification curves and corresponding relative fluorescence intensities are shown for each condition. “*” indicates statistically significant differences (*p* < 0.05), and “ns” indicates no significant difference.

For the *V. vulnificus* detection system, the optimized conditions were identified as 1.0 μM gDNA, 0.5 mM Mn^2+^, and 6 μL PfAgo protein, based on the highest fluorescence intensities observed under these conditions (Figure 5a–c).

### 3.3. Sensitivity and Specificity Evaluation

The specificity and sensitivity of the optimized LAMP-PfAgo systems for *V. alginolyticus* and *V. vulnificus* were evaluated using target and non-target bacterial strains.

**Figure 6:**
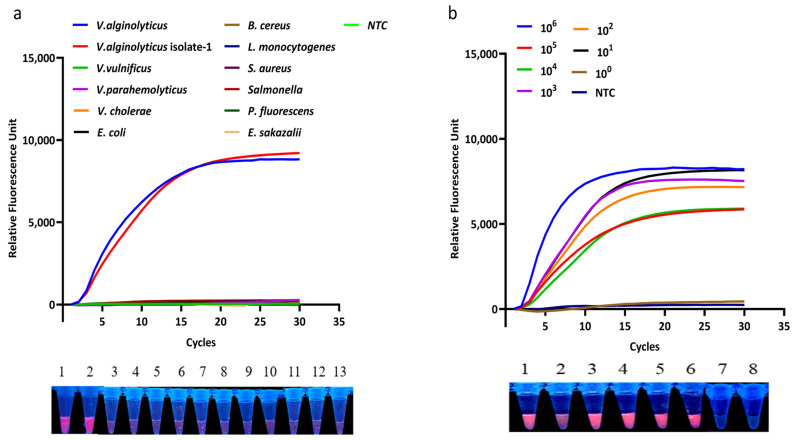
Specificity and sensitivity evaluation of the *V. alginolyticus* LAMP-PfAgo detection system. (**a**) Specificity analysis using *V. alginolyticus* strains and non-target bacterial species. (**b**) Sensitivity evaluation using 10-fold serial dilutions of *V. alginolyticus* bacterial suspensions.

For the *V. alginolyticus* detection system, strong fluorescence signals were observed only for the standard strain and isolates of *V. alginolyticus*, whereas non-target bacteria produced weak signals and were judged negative (Figure 6a). These results indicate that the assay specifically detected the tested *V. alginolyticus* strains within the bacterial panel used in this study. In the sensitivity assay, positive fluorescence signals were detected in bacterial suspensions ranging from 3 × 10^6^ to 3 × 10^1^ CFU·mL^−1^, whereas the fluorescence intensity at 3 × 10^0^ CFU·mL^−1^ did not differ significantly from the negative sample (Figure 6b). The LOD of the *V. alginolyticus* LAMP-PfAgo assay was determined to be 3 × 10^1^ CFU·mL^−1^.

**Figure 7:**
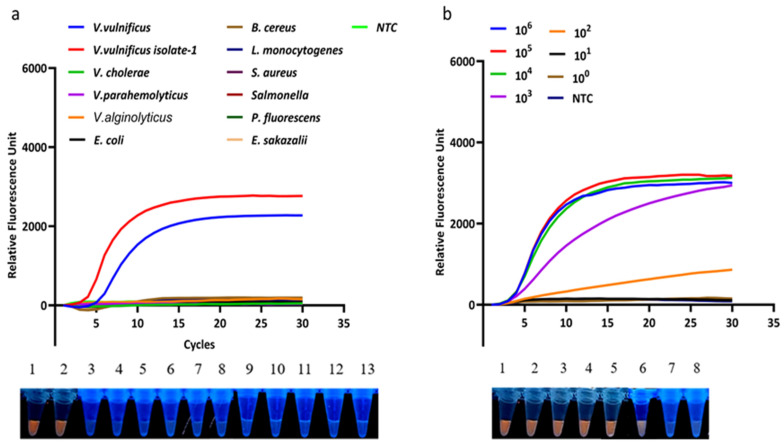
Specificity and sensitivity evaluation of the *V. vulnificus* LAMP-PfAgo detection system. (**a**) Specificity analysis using *V. vulnificus* strains and non-target bacterial species. (**b**) Sensitivity evaluation using 10-fold serial dilutions of *V. vulnificus* bacterial suspensions.

The *V. vulnificus* detection system exhibited specific detection of the tested *V. vulnificus* strains, with no obvious fluorescence signals observed for the non-target bacteria included in this study (Figure 7a). In the sensitivity evaluation, the LOD of the *V. vulnificus* LAMP-PfAgo assay was 10^2^ CFU·mL^−1^ (Figure 7b).

### 3.4. Artificial Contamination Assay

**Figure 8:**
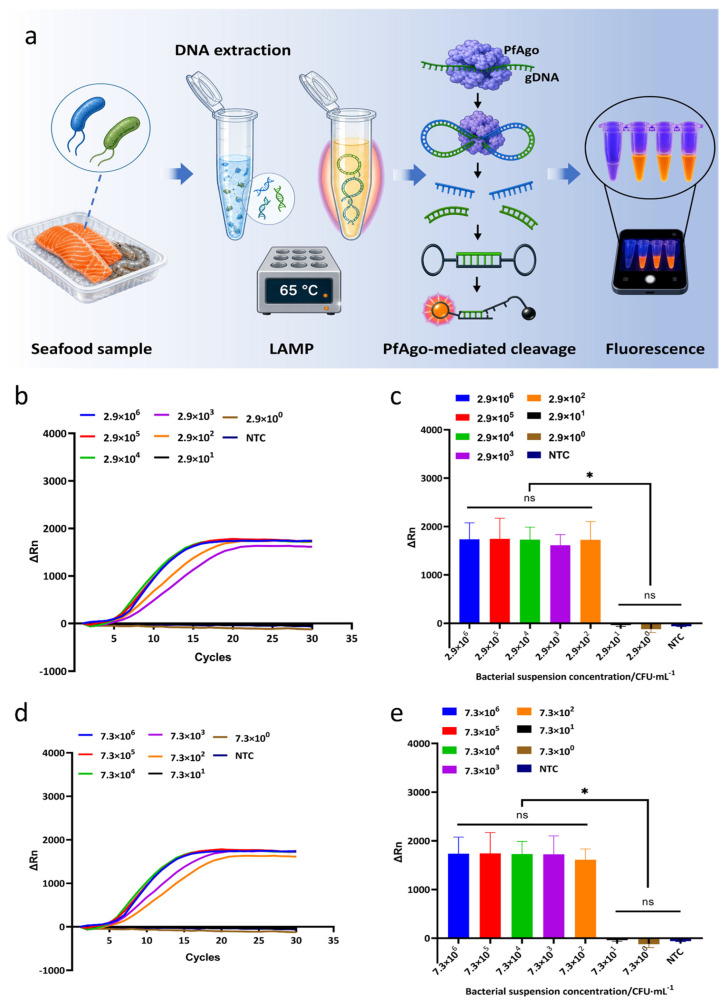
Detection of artificially contaminated salmon samples using the LAMP-PfAgo assay. (**a**) Schematic overview of the assay workflow, including DNA extraction from seafood samples, LAMP at 65 °C, PfAgo-mediated guide-directed cleavage at 95 °C and fluorescence-based result interpretation. (**b**) Real-time fluorescence curves for salmon samples spiked with *V. alginolyticus*. (**c**) Endpoint fluorescence intensities of the corresponding *V. alginolyticus* reactions. (**d**) Real-time fluorescence curves for salmon samples spiked with *V. vulnificus*. (**e**) Endpoint fluorescence intensities of the corresponding *V. vulnificus* reactions. NTC, no-template control. “*” indicates statistically significant differences (*p* < 0.05), and “ns” indicates no significant difference. Note: The bacterial concentrations shown in panels (**b**–**e**) refer to the final concentrations in the salmon/APW homogenates after addition of bacterial suspensions to salmon samples. The bacterial suspensions used for spiking were approximately 100-fold more concentrated than the final homogenates.

To assess the applicability of the LAMP-PfAgo assay in a food matrix, salmon samples artificially contaminated with *V. alginolyticus* or *V. vulnificus* were analyzed without enrichment culture after boiling-based DNA extraction (Figure 8a). For *V. alginolyticus*, real-time fluorescence curves increased rapidly for samples prepared with final contamination levels from 2.9 × 10^6^ to 2.9 × 10^2^ CFU·mL^−1^ in the sample homogenate, whereas lower concentrations remained at background levels (Figure 8b). Endpoint fluorescence analysis further separated the positive groups from the negative controls, with robust ΔRn values retained at the 2.9 × 10^2^ CFU·mL^−1^ level (Figure 8c). Similar results were obtained for *V. vulnificus*. Positive fluorescence curves were observed for salmon samples prepared with final contamination levels from 7.3 × 10^6^ to 7.3 × 10^2^ CFU·mL^−1^ in the sample homogenate, while the 7.3 × 10^1^ and 7.3 × 10^0^ CFU·mL^−1^ groups remained comparable to the no-template control (Figure 8d). Endpoint fluorescence analysis confirmed that the positive groups were distinguishable from the negative controls down to 7.3 × 10^2^ CFU·mL^−1^ (Figure 8e). These results indicate that the LAMP-PfAgo assay can detect both *V. alginolyticus* and *V. vulnificus* in salmon samples under the tested conditions. For both pathogens, the practical detection limit was approximately 10^2^ CFU·mL^−1^ in the final homogenate.

## 4. Discussion

This study establishes a two-step LAMP-PfAgo workflow that couples rapid isothermal amplification with Argonaute-mediated sequence verification for the detection of *V. alginolyticus* and *V. vulnificus*. LAMP provides high-yield amplification under constant temperature, whereas PfAgo adds a programmable cleavage step guided by short gDNAs. In this assay, molecular beacon cleavage is mediated by target-derived secondary guide fragments generated after PfAgo cleavage of the LAMP amplicons. This two-layer recognition strategy is well suited to *Vibrio* detection, where closely related species and nonspecific amplification can complicate interpretation.

*V. alginolyticus* and *V. vulnificus* are both important marine and seafood-associated pathogens, but their high relatedness to other *Vibrio* species can make species-level identification difficult [6]. Culture-based identification remains reliable but is time-consuming, whereas conventional PCR shortens detection time but still requires thermal cycling instrumentation and laboratory infrastructure, with reported detection limits typically around 10^2^–10^3^ CFU mL^−1^ [23]. Although traditional LAMP assays are sensitive, their reliance on nonspecific signal readouts can lead to false-positive results [8]. The PfAgo-mediated secondary recognition step used here addresses this limitation by adding sequence verification after LAMP, thereby improving confidence in positive signals while preserving the simplicity of a two-step workflow. Consistent with this design, the assay achieved a detection limit of 3 × 10^1^ CFU mL^−1^ for *V. alginolyticus* and remained detectable in salmon samples without enrichment, with a practical detection limit of 2.9 × 10^2^ CFU mL^−1^ in the final sample. For *V. vulnificus* detection, Tian et al. established a visual LAMP assay targeting the *gyrB* gene, with a reported analytical sensitivity of 10 fg·μL^−1^ genomic DNA and a 30 min reaction at 65 °C [24]. Yang et al. developed an RPA-lateral flow strip assay for raw seafood that completed detection within 35 min at 37 °C, with a sensitivity of 2 copies or 10^−1^ CFU per reaction; in spiked seafood samples, the method detected 1 CFU per 10 g after enrichment [25]. A concise comparison of the present assay with representative nucleic acid detection methods is provided in Table 6. Compared with these assays, the present LAMP-PfAgo method retains the rapid and simple features of isothermal amplification, while introducing PfAgo-mediated sequence verification after amplification. This additional recognition step provides stronger target confirmation and may reduce false-positive signals caused by nonspecific amplification, which is advantageous for species-level identification of closely related *Vibrio* pathogens.

Compared with CRISPR-Cas-based detection systems, the LAMP-PfAgo assay offers a complementary route with flexible target design. LAMP-CRISPR/Cas12a assays can achieve extremely low detection limits [30], but Cas12a recognition depends on PAM sequences and requires careful reaction optimization [11]. In addition, Cas13a-based assays require an additional transcription step, which increases assay complexity and time [31]. In contrast, PfAgo does not require PAM sequences and uses short, stable gDNAs for target recognition [13]. Previous studies have shown that Ago-based detection systems combined with isothermal amplification can reach detection limits as low as 6.68 CFU·mL^−1^ [32], supporting the broader feasibility of this strategy.

The specificity results further support the value of the PfAgo verification step. The developed assay distinguished the tested target *Vibrio* species from the non-target bacteria included in this study. This is important because high genetic similarity among Vibrio species can cause misidentification in some molecular assays [33]. The PfAgo-mediated sequence-specific cleavage therefore helps compensate for the nonspecific amplification risk inherent to LAMP [14]. *V. cholerae* and *V. parahaemolyticus* are also major seafood-associated pathogens with greater recognized importance in human disease. In the present study, they were included as non-target *Vibrio* species for specificity evaluation, whereas *V. alginolyticus* and *V. vulnificus* were selected as target organisms to evaluate the LAMP-PfAgo strategy. Although the specificity panel included *V. parahaemolyticus* and *V. cholerae* as seafood- and foodborne-associated non-target *Vibrio* species, other closely related species, such as *V. fluvialis*, *V. mimicus*, and *V. harveyi*, were not included in the present study. In addition, *V. vulnificus* biotype information was not fully evaluated in the present study. Therefore, further validation using defined biotype 1 and biotype 2 strains is needed before extending the assay claim to all *V. vulnificus* biotypes. Including these species in future studies would further strengthen the specificity evaluation and support broader validation of the assay using closely related *Vibrio* species and foodborne or environmental isolates.

The artificial contamination experiment demonstrated that the assay can detect both *V. alginolyticus* and *V. vulnificus* in salmon samples with a practical LOD of approximately 10^2^ CFU·mL^−1^ in the final sample homogenate under the tested conditions. This workflow is faster than conventional culture-based methods, which often require 24–72 h [23], and may be more suitable for rapid screening in food safety monitoring. However, performance in additional seafood matrices and field settings remains to be evaluated.

Overall, the LAMP-PfAgo assay combines LAMP-based isothermal amplification with sequence-specific Argonaute cleavage. Within the tested sample types and bacterial panels, this strategy improved analytical specificity while maintaining practical sensitivity, supporting its further development for on-site foodborne pathogen detection.

## 5. Conclusions

This study demonstrates a two-step LAMP-PfAgo detection strategy for *V. alginolyticus* and *V. vulnificus* that combines rapid LAMP with guide-directed Argonaute cleavage. The assay achieved specific detection of the tested target strains with limits of detection of 3 × 10^1^ CFU·mL^−1^ for *V. alginolyticus* and 10^2^ CFU·mL^−1^ for *V. vulnificus*. In artificially contaminated salmon samples, the practical detection limits were 2.9 × 10^2^ CFU·mL^−1^ for *V. alginolyticus* and 7.3 × 10^2^ CFU·mL^−1^ for *V. vulnificus* in the final homogenates. These results support LAMP-PfAgo as a practical route for sequence-verified seafood pathogen screening, while further work is needed to validate broader matrices, closed-tube operation, and multiplex detection.

## Figures and Tables

**Table 1:** Bacterial strains used in this study.

Bacterial	Strain No.
*V. alginolyticus*	ATCC17749
*V. alginolyticus*	VA Isolates-1
*V. vulnificus*	ATCC 27562
*V. vulnificus*	VV Isolates-1
*V. cholerae*	CICC 23794
*V. parahaemolyticus*	CICC 21528
*Pseudomonas fluorescens*	CICC 21620
*Bacillus cereus*	CMCC 63301
*Enterobacter sakazakii*	CICC 21563
*Escherichia coli*	CICC 21530
*Listeria monocytogenes*	CICC 21662
*Staphylococcus aureus*	CICC 22942
*Salmonella enteritidis*	CICC 21482

**Table 2:** LAMP primer sequences designed for *V. alginolyticus*.

Primer Name		Sequences (5′→3′)
VA-614	F3	AGTTAATCAAGCCGCCTCAG
	B3	ACCCGAGCTCACAACCTC
	FIP	TAGAGCAAAGTAAGCTCGCTCGCAAAACGTTGCGCGTGAGTT
	BIP	TTAGAAACTGCCCTTCCCGAAGCTCTTAATTGGCTCGCTGTACG
	LF	TTGCATTGCTTAGTTGGC
	LB	CGTTTAAGCGGTGAAATAGG
VA-14	F3	AGTTAATCAAGCCGCCTCAG
	B3	ACCCGAGCTCACAACCTC
	FIP	TAGAGCAAAGTAAGCTCGCTCGCAAAACGTTGCGCGTGAGTT
	BIP	TTAGAAACTGCCCTTCCCGAAGCTCTTAATTGGCTCGCTGTACG
VA-28	F3	ACTGACTGGCTCGCTCAC
	B3	GGACGCAACCAACGATGA
	FIP	CGTCCGGCGATTTCTTCCAAACCAACCCTTCACGCTACCG
	BIP	GCTATGGTTCATGGTGGACCCTGCCCCGACAGATGTTGAG
	LB	ATCCGGCTTCAACCAATTCTGC
VA-618	F3	ATCGCAATTGGCCCTGTC
	B3	TGCAATAGCGTGAAGATGGC
	FIP	GTGCCGAAGCCGTATCACCACCCGTGTTTGGTGCGTCTA
	BIP	CCACCCTGGCACCAGTCAACGCGGTAAGCACTCTTTCTCA
	LF	CGTAGAAAATGCGAGCGGAAAAT
	LB	CAGAGTGTATTGAGGCTGCAC

**Table 3:** LAMP primer sequences designed for *V. vulnificus*.

Primer Name		Sequences (5′→3′)
VV-51	F3	ACTGCGAGTGGTTTCCATC
	B3	GCTCTCTGGTGAAGCAAGAA
	FIP	CGCCGGATACGTACCAAAGTGATCTACCATCACTTGCTTGGC
	BIP	AACTTGCTACCGAGACCCGCCCGGCTGAAATCGATCTCAT
	LF	TGAAGCATGGCCTTTTTGGC
VV-1	F3	ACTGCGAGTGGTTTCCATC
	B3	GCTCTCTGGTGAAGCAAGAA
	FIP	CGCCGGATACGTACCAAAGTGATCTACCATCACTTGCTTGGC
	BIP	AACTTGCTACCGAGACCCGCCCGGCTGAAATCGATCTCAT
VV-512	F3	CTGCGAGTGGTTTCCATCTC
	B3	TTCCTCTGTACTGGCTCTCT
	FIP	TTAAGCTGCGCTTTTTCGCCGCTTGGCTCACCCGACTCA
	BIP	CCGCTGAAGCATGGCCTTTTTGGGTGAAGCAAGAATCCCCG
	LF	GCAAAAGATCACTCGTGATGAGAT
VV-12	F3	CTGCGAGTGGTTTCCATCTC
	B3	TTCCTCTGTACTGGCTCTCT
	FIP	TTAAGCTGCGCTTTTTCGCCGCTTGGCTCACCCGACTCA
	BIP	CCGCTGAAGCATGGCCTTTTTGGGTGAAGCAAGAATCCCCG

**Table 4:** gDNA and probe sequence for *V. alginolyticus*.

Primer Group		Sequences (5′→3′)
VA-gDNA1+2	VA-gDNA1	GGTATGGCCTTTTACA
	VA-gDNA2	ATCACAGGACAGCCAG
VA-gDNA3+4	VA-gDNA3	ATGGCCTTTTACAATC
	VA-gDNA4	ACAGGACAGCCAGCTG
VA-gDNA5+6	VA-gDNA5	CGGTATGGCCTTTTAC
	VA-gDNA6	AATCACAGGACAGCCA
MB	VA-MB	5′ 6-ROX-ATGGCCTTTTACAATCACAGGACAGCCAGCT-3′ BHQ-2

Note: All gDNAs were synthesized with a 5′-phosphate modification.

**Table 5:** gDNA and probe sequence for *V. vulnificus*.

Primer Group		Sequences (5′→3′)
VV-gDNAs1+2	VV-gDNA1	AAAGCGCAGCTTAATC
	VV-gDNA2	ACACGTTACCACTCAA
VV-gDNAs3+4	VV-gDNA3	AAGCGCAGCTTAATCA
	VV-gDNA4	CACGTTACCACTCAAT
VV-gDNAs5+6	VV-gDNA5	AGCGCAGCTTAATCAC
	VV-gDNA6	ACGTTACCACTCAATA
MB	VV-MB	5′-6-NED-AAGCGCAGCTTAATCACACGTTACCACTCA-3′BHQ2

Note: All gDNAs were synthesized with a 5′-phosphate modification.

**Table 6:** Comparison of the present LAMP-PfAgo assay with previously reported nucleic acid detection methods for related foodborne pathogens.

Target	Method	LOD	Assay Time	Equipment/Readout	Sample	Reference
*V. alginolyticus* *V. vulnificus*	LAMP-PfAgo	3 × 10^1^ CFU·mL^−1^ for *V. alginolyticus*, 10^2^ CFU·mL^−1^ for *V. vulnificus*, 2.9 × 10^2^ CFU·mL^−1^ in the final sample for *V. alginolyticus* and 7.3 × 10^2^ CFU·mL^−1^ in the final sample for *V. vulnificus*	40 min LAMP and 30 min PfAgo cleavage	Constant-temperature amplification device plus fluorescence readout	Bacterial suspensions, spiked salmon	This study
*V. vulnificus*	Visual LAMP	10 fg uL^−1^ genomic DNA	30 min at 65 °C	Water bath or heating block, visual readout	Aquatic products and environmental water	[24]
*V. vulnificus*	RPA-LFS	2 genome copies or 10^−1^ CFU per reaction, 1 CFU/10 g in spiked seafood after enrichment	35 min at 37 °C	Low-temperature incubator plus lateral flow strip	Raw seafood	[25]
*V. vulnificus*	RAA-CRISPR/Cas12a	2 copies per reaction	40 min	Isothermal amplification plus UV/fluorescence visual readout	Spiked blood, stool and shrimp samples	[26]
*V. cholerae* *V. vulnificus*	Duplex RPA-LFS	10^1^ gene copies per reaction, 1 CFU/10 g in spiked food	30 min at 37 °C and 5 min strip visualization	Low-temperature incubator plus three-segment lateral flow strip	Spiked shrimp and clinical samples	[27]
*V. vulnificus*	RAA combined with dual-target test strip	10 CFU·mL^−1^ for *gyrB* and 100 CFU·mL^−1^ for *vvhA*	Within 50 min including sample preparation	Low-temperature isothermal amplification plus test strip/smartphone grayscale analysis	Oyster, fish and shrimp	[28]
*V. alginolyticus*	RPA-CRISPR/Cas13a-LFD	10 copies uL^−1^	<50 min	Isothermal amplification plus lateral flow dipstick	Bacterial isolates and infected mouse blood	[7]
*E. coli* O157:H7 *S. aureus*, *S. typhimurium* *C. sakazakii*	Endo V-activated PfAgo detection (VPN)	10^1^ CFU·mL^−1^ for four foodborne pathogens	RPA/Endo V step plus 40 min PfAgo cleavage	Isothermal amplification plus fluorescence readout	Beef and milk samples	[29]

Note: LOD values are reported as described in the cited studies and are not directly interchangeable because template types, sample preparation procedures and matrix validation differed among assays. RPA, recombinase polymerase amplification; RAA, recombinase-aided amplification; LFS/LFD, lateral flow strip/dipstick.

## Data Availability

The original contributions presented in the study are included in the article; further inquiries can be directed to the corresponding author.

## Abbreviations

The following abbreviations are used in this manuscript:

gDNA	guide DNA
I/O	inner-to-outer primer ratio
LAMP	loop-mediated isothermal amplification
LOD	limit of detection
NTC	no-template control
PfAgo	Pyrococcus furiosus Argonaute
